# The smectic order of wrinkles

**DOI:** 10.1038/ncomms15809

**Published:** 2017-07-18

**Authors:** Hillel Aharoni, Desislava V. Todorova, Octavio Albarrán, Lucas Goehring, Randall D. Kamien, Eleni Katifori

**Affiliations:** 1 Department of Physics and Astronomy, University of Pennsylvania, Philadelphia, Pennsylvania 19104, USA; 2 Max Planck Institute for Dynamics and Self-Organization (MPIDS), 37077 Göttingen, Germany; 3 School of Science and Technology, Nottingham Trent University, Nottingham NG11 8NS, UK

## Abstract

A thin elastic sheet lying on a soft substrate develops wrinkled patterns when subject to an external forcing or as a result of geometric incompatibility. Thin sheet elasticity and substrate response equip such wrinkles with a global preferred wrinkle spacing length and with resistance to wrinkle curvature. These features are responsible for the liquid crystalline smectic-like behaviour of such systems at intermediate length scales. This insight allows better understanding of the wrinkling patterns seen in such systems, with which we explain pattern breaking into domains, the properties of domain walls and wrinkle undulation. We compare our predictions with numerical simulations and with experimental observations.

## Abstract (web summary)

A thin elastic sheet can develop wrinkles which arrange into patterns similar to those characteristic of liquid crystals. Here the authors use this analogy to propose a mapping between the elastic sheet problem and the smectic liquid crystal problem which can enable a better understanding of wrinkling.

The Gauss–Bonnet theorem is the bane of all mapmakers: it is impossible to represent a spherical surface on a flat plane without distorting areas or angles^1^. Typically, we concern ourselves with how a flat drape or sheet can be used to wrap a curved object—wrinkles^2^, folds and creases^3^ occur that can, by a sequence of careful pleats (origami)^4^ or excisions (kirigami)^5^, be neatly hidden or removed. This works similarly in the reverse situation. Suppose we take half a beach ball and try to lay it flat on the table. Though the cap could elastically contract or stretch to become completely flat, our experience is that a thin surface will buckle into the third dimension into patterns of wrinkles and folds.

Wrinkling allows regions that are under lateral compression to accommodate their excess length while avoiding significant in-plane contraction. When put on a bulk of liquid, a natural length scale for such wrinkling arises from the competition between elastic bending (which tends to prefer smoother long-wavelength wrinkles) and gravity (which pushes towards low-amplitude wrinkles). The emergent wavenumber in such a system is 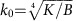 where *B* is the bending modulus of the thin sheet and *K* is the substrate-response modulus^6^,^7^. This relation has been well studied and was generalized to cases where the substrate is not liquid but rather an elastic solid or, more generally, any wavelength-dependent retracting force^8^. It was also generalized to cases where an effective retracting force emerges from tangential elastic tension or from substrate curvature along the wrinkle direction^9^,^10^, and was shown to hold locally even when those fields vary across the system^10^. In contrast, in systems without such preferred local wavelength, for example, the ‘torn garbage bag’ problem^11^,^12^, one finds solutions which contain a wide cascade of wavelengths spanning from scales of the non-Euclidean geometry down to the thickness scale (or, for an infinitely thin sheet, all the way down^13^).

In the following we focus on systems in the regime where there is a scale separation between the thickness of the sheet, the periodicity 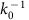 and the frustration length scale—either *D*, the lateral size of the elastic shell, or *R*, the smallest radius of curvature appearing in the shell’s reference metric. If *Y*, *B* and *K* are the stretching, bending and substrate moduli, respectively, then this length scale separation reads 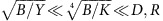. Equivalently, this can be written in terms of the Föppl–von Kármán number *γ*=*YD*^2^/*B* (or *YR*^2^/*B*) and a stretchability parameter 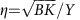 as 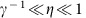. In this work we show that such systems behave like 2D smectics at intermediate length scales, while behaving elastically at large length scales to satisfy global geometric constraints. This elastic/smectic analogy allows better understanding of the emergent wrinkle patterns, which can be faithfully interpreted in terms of grain boundaries^14^,^15^, dislocations and possibly even focal conic domains^16^ (Fig. 1). We demonstrate this fact by explaining several features of those patterns, both qualitatively and quantitatively. This analogy also opens a door for efficient numerical study of these systems; the same scale separation that poses a problem for conventional elastic simulations may allow more efficient numerics via our smectic description.

## Results

### Energy of wrinkled patterns

Before introducing the physical model, we must start with choosing a convenient coordinate system to represent the configuration of a thin wrinkled sheet. Such representation must be able to account for long-wavelength deformations to capture the overall geometry and, as we shall see, short wavelength wrinkles that take up the excess area. For this purpose we choose surface coordinates in which the configuration, assumed to be lying near the plane *z*=0, takes the form


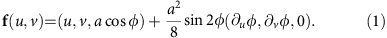


where *a* and *ϕ* are scalar fields that in general depend on *u* and *v*. While this choice of Cartesian-like coordinates {*u*,*v*} may seem unintuitive, it can be rationalized by considering a uniform wrinkle pattern. Let the amplitude *a*(*u*,*v*) and wavenumber ∇*ϕ*(*u*,*v*) be constant, that is, *a*(*u*,*v*)=*a* and *ϕ*(*u*,*v*)=*qu*. Then the arclength of a curve along *u* is just


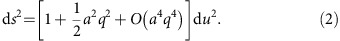


When the amplitude is small 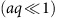, d*s*/d*u* is approximately constant while still capturing the overall excess length taken up by the wrinkle. With the representation in equation (1), slow variations in *a* and ∇*ϕ* do not alter the local wrinkle features. Calculations in terms of *u* and *v* are greatly simplified because the baseline, wrinkled metric does not have a short wavelength dependence on *u*, *v*. This is the major advantage of using these coordinates, and the reason for introducing the *O*(*a*^2^) correction to equation (1). In addition, it is clear from equation (1) that for small amplitudes the coordinates {*u*, *v*} are close to the Cartesian {*x*, *y*} coordinates of the projection of **f** onto the plane.

We will subsequently assume that the amplitude *a*(*u*,*v*) and phase *ϕ*(*u*,*v*) represent a slowly varying envelope of the wrinkled pattern, namely 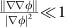 and 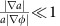. We further assume small amplitude wrinkles, namely the excess length to be 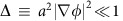. These assumptions directly relate to properties of the solution rather than to controlled parameters in the problem, but we will later show that they are consistent with results obtained from the model in a wide parameter regime and with solutions observed in experiments and simulations. In the following text we use the ∼ notation to indicate equality up to higher order terms in one or more of these small parameters.

With the above assumptions, the 2D metric and curvature tensors of configuration (1) take the form


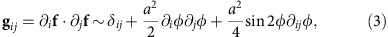



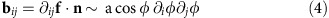


with *i*, *j*∈{*u*,*v*}, and **n** the unit vector normal to the surface. Keeping our assumed scale separation in mind, we will be interested in looking at coarse-grained versions of different fields, namely ones which are averaged over oscillations at the wrinkle length scale |∇*ϕ*|^−1^, and have only features which persist across at least several wrinkles. For that purpose we define a coarse-graining operator 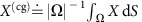, where Ω is a region of radius ≳|∇*ϕ*|^−1^ around each point and *X* can be any tensor field. Scale separation renders this definition unambiguous; the sheet’s reference metric (and of course the planar metric) can be approximated as Euclidean at the scale of Ω (since rad(Ω)<<*D*) and therefore integration, even of tensor fields, is well-defined. Since on the scale of Ω the envelope of *a*, ∇*ϕ* can be considered constant, while *ϕ* itself changes significantly, this coarse-graining operation will be manifested in the vanishing of all high-frequency modes ∝e^*inϕ*(*u*,*v*)^, where *n*≥1, in *X*. For tensor fields this can be done element-wise in coordinates. Applying this to the metric (3) and curvature (4) tensors yields


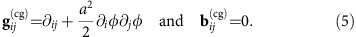


Let us now assume a scenario in which a thin elastic non-Euclidean shell is put atop a bulk of liquid (Fig. 2). The system energy *H* takes contributions from both the elastic energy of the thin sheet—stretching and bending^17^,^18^—and from the gravitational potential energy associated with displacing liquid elements (perfect wetting of the sheet is assumed). It can therefore be written in the form


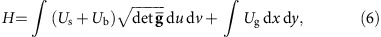


where


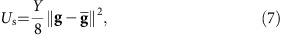



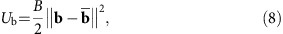



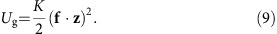


For an isotropic material the stretching modulus is 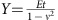 (with *E* Young’s modulus, *t* the thickness and *ν* Poisson’s ratio), bending modulus is 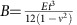, and substrate modulus is *K*=*ρg* (fluid density and gravitational acceleration, respectively). The parameters 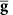 and 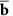 are the shell’s reference metric and curvature tensors, respectively, and 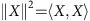 where 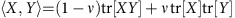 and tr denotes contraction with the contravariant reference metric tensor 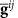. Note that the gravitational potential is not proportional to 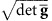, and that it is not fully determined by the surface’s fundamental forms, because of the non-covariance of the liquid (in the 2D geometry). The difference between its proper area form d*x* d*y* and our coordinate area form d*u* d*v* is high-order in our small parameters and will be neglected in the following. Substituting equation (5) into equations (7)–(9) yields


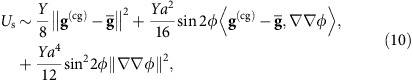



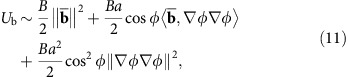



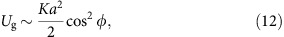


where (∇∇*ϕ*)_*ij*_=*∂*_*ij*_*ϕ* and (∇*ϕ*∇*ϕ*)_*ij*_=*∂*_*i*_*ϕ∂*_*j*_*ϕ* (it is worth noting that 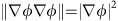). Under our assumed scale separation, the Hamiltonian (6) (or any large-scale integral) is unchanged by coarse-graining its integrand, thus we can rewrite equations (6) and (10)–(12), 29, 30:


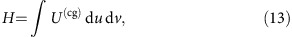


where


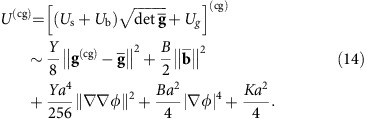


With our thinness and slowly varying envelope assumptions, the modulus *Y* that appears in the first term of equation (14) is much larger than the other moduli in equation (14). It follows that 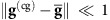, hence that the value of the local excess length Δ=*a*^2^|∇*ϕ*|^2^ is nearly constrained locally (see Supplementary Note 1 for a detailed derivation). Under this constraint, the bending and gravity terms in equation (14) are minimized at the well-studied finite wavenumber 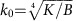, around which we can expand these terms.

### The elastic/smectic energy functional

We now rearrange the energy density (14) and write it as a sum of two new components:


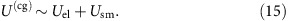


The first component in equation (15), which we denote the elastic term, takes the form


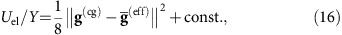


where we find an effective reference metric, 
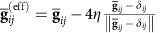
, that is slightly shifted from the actual reference metric of the shell towards a flat Euclidean metric according to the stretchability parameter *η*. The elastic energy component (16) penalizes deviations of the coarse-grained metric from this effective reference metric.

To illustrate the nature of this energy component, it is helpful to consider a wrinkled configuration of constant amplitude *a*. The coarse-grained metric (5) of such a configuration is identical to that of a surface whose topographic map is given by the wrinkled pattern, where wrinkles represent level-sets spaced 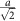 in the *z* direction. Therefore, equation (16) penalizes deviations of that smooth surface’s geometry from the target geometry 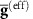. However, this geometry is the coarse-grained version of many different wrinkle patterns, for example, taking a topographic map of the same surface from different projection angles or with a different choice of level-set spacing (wrinkle amplitude).

This degeneracy is broken by the second component in equation (15), the smectic term, which takes the form


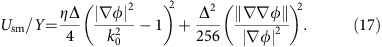


The first term, smectic compression, penalizes deviations of wrinkle spacing from the preferred wavenumber *k*_0_. Its modulus comes from the bending-gravity interplay, but is also proportional to the local excess length Δ which may therefore be thought of as a (perhaps spatially varying) local smectic order parameter. The second term, smectic curvature, penalizes wrinkle bending (Fig. 2d). Interestingly it has nothing to do with elastic bending, and actually comes only from residual tangent-to-sheet elastic stretching. A geometric intuition can be given to this term—unlike straight wrinkles, bent wrinkles have nontrivial Gaussian curvature which oscillates at half the wrinkle wavelength. If the target geometry is smooth at the wrinkle scale, that implies strain which results in elastic stretching, which is the source for the second term in (equation 17).

A very similar analysis can be performed when the substrate is soft elastic solid rather than liquid; the wrinkle wavenumber *k*_0_ is then to be replaced with 
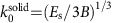
, where *E*_s_ is the substrate’s Young’s modulus and the substrate’s Poisson’s ratio is assumed 0.5 (ref. 8). The relevant dimensionless stretchability parameter *η* is 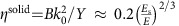, which does not depend on the thickness (as is expected from dimensional analysis). The following results hold in either physical scenario.

We next explore some of the outcomes of this analogy between wrinkles and smectics. to test our predictions, we employ two-dimensional (2D) finite-element simulations (Abaqus) of an elastic spherical shell with bulk gravitational force. The elastic and gravitational moduli, as well as the sheet geometry, can be tweaked to give us full control over the relevant dimensionless parameters (*η*, *γ*). Fluid surface tension exists in the experiment and is neglected in the simulation, however, in the relevant parameter regime its effect is negligible (Supplementary Fig. 2). Figure 2c,d demonstrates the good agreement in equilibrium configurations between the simulation and an experiment of a PDMS shell on water, performed with the same physical parameters. An extensive experimental and numerical research of such systems is in progress.

### Domain walls

From the two smectic moduli in equation (17), one can extract a smectic penetration length


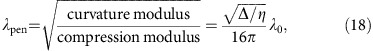


where *λ*_0_ is the wrinkle wavelength. The penetration length *λ*_pen_ is a typical length over which smectic layers—or wrinkles—may bend smoothly. Hence, the wall between two smectic domains, with an angle difference 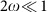 between their wrinkle directions, will be of typical width 2*λ*_pen_/tan *ω* (ref. 19). From equation (18) one learns that domain wall width is proportional to 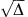, hence proportional to the amplitude. In systems with a non-Euclidean underlying geometry, the amplitude may vary across the system (since 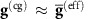). Consequently domain walls may vary in width, possibly even along one continuous wall, as can be well seen in Fig. 3a. Remarkably, equation (18), based entirely on the theory of smectic liquid crystals, provides a precise prediction for the relation between the width of domain walls and local amplitude in wrinkled patterns. This prediction is in excellent agreement with measurements taken on our wrinkled elastic sheet simulations (Fig. 3c).

As Fig. 3 suggests, the above prediction appears to be valid only above certain values of the parameter *η*. Nonetheless, its breakdown is also explained by smectic theory; indeed, many systems that exhibit layered patterns, ranging from diblock copolymer melts^15^,^20^,^21^ to Rayleigh–Bénard convection^22^,^23^, show a transition from ‘Chevron’-shaped domain walls at small wall angles to Ω-shaped walls at large angles. This bifurcation is achieved via the formation of a pair of disclinations at each layer, to better maintain layer spacing in the system. A similar transition was observed, though to the best of our knowledge not explicitly discussed, in wrinkled herringbone patterns^24^,^25^, seemingly via the formation of d-cones (ref. 26). The critical domain boundary angle for this transition is made wider when the thickness of the sheet (or equivalently *η*) increases, thus not allowing focusing of the curvature into small d-cones cores. Since the width of the Ω-shaped domain wall scales as the wrinkle wavelength *λ*_0_, and not as the penetration length, one expects domain walls to abruptly change their scaling with decreasing *η*, as seen in Fig. 3. For wider domain wall angles, the Chevron-type walls and consequently the width ∝*λ*_pen_ relation are expected to persist onto narrower sheets.

### Undulation instability

In smectic systems, certain boundary conditions may induce an undulation instability in the smectic structure^27^,^28^. At its onset, the wavelength of this undulation is


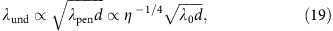


where the *d* is a system scale at which boundary conditions are introduced. To test if similar behaviour exists in wrinkled systems, we designed a numerical experiment (Fig. 4) that follows the footsteps of ref. 27 in their smectic-A experiment. In our simulations, we hold a thin strip at its boundaries at a wavelength *λ*_B_ which is slightly mismatched with the preferred wrinkle spacing *λ*_0_=0.95*λ*_B_. One resolution for such mismatch may be an undulation, which allows layer spacing in the bulk to be smaller than on the boundary, while paying some cost for layer bending. The predicted optimum for this exchange is given by equation (19). We examine a sequence of systems with fixed geometry and wrinkle wavelength, however, with penetration/undulation lengths varying between simulations (achieved via changing both fluid density and sheet thickness so that *λ*_0_ remains constant, however, *η* changes).

Indeed, an undulation in the wrinkling pattern appears (Fig. 4a). The observed undulation wavelength scales as the square root of the penetration length (Fig. 4b), as accurately predicted by equation (19). When using the ribbon width as a surrogate for the system scale *d*, we get a factor of ≈1.7 between the observed wavelength and the predicted one, asserting that ribbon width may not be the relevant system scale.

**Figure 1:**
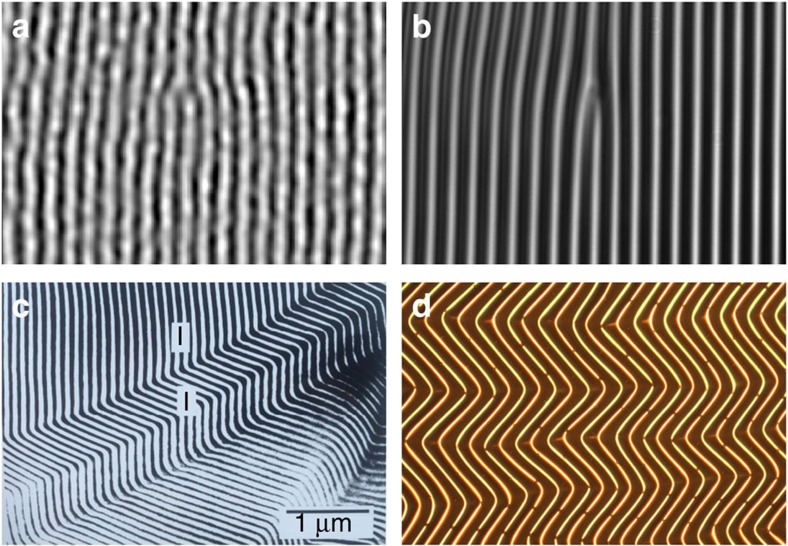
Smectic patterns in wrinkles. Dislocations (**a**,**b**) and Herringbone patterns (**c**,**d**) in diblock copolymer melts (**a**,**c**) and in thin sheets on a soft elastic substrate (**b**,**d**). (**a**) reprinted with permission from ref. 33, copyright (2002) by the American Physical Society; (**b**) reprinted from ref. 30, with the permission of AIP Publishing, high-resolution version courtesy of S. Yang; (**c**) reprinted with permission from ref. 15, copyright (1994) American Chemical Society, high-resolution version courtesy of S. Gido; (**d**) courtesy of J.A. Rogers.

**Figure 2:**
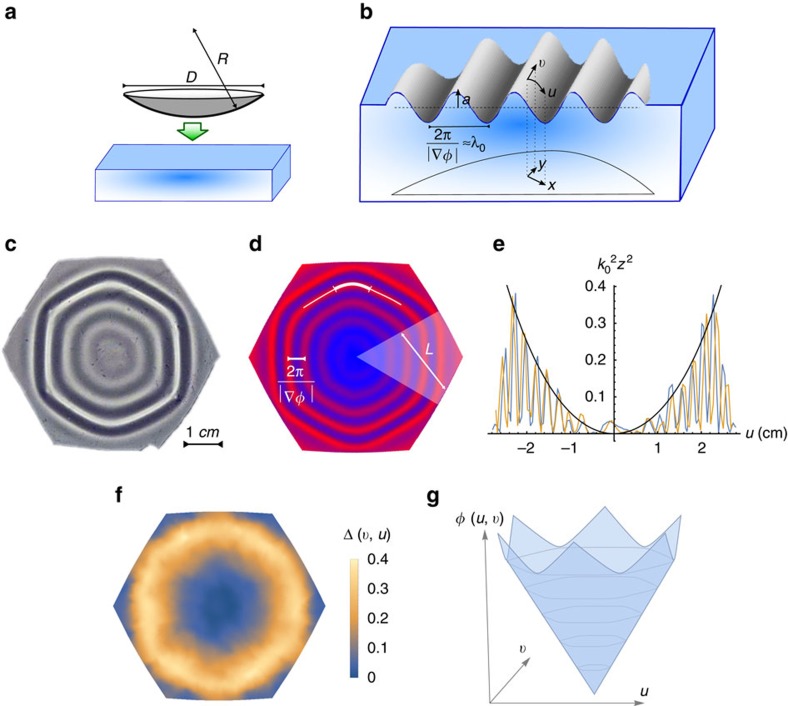
Non-Euclidean shells on a Euclidean substrate. (**a**) A spherical shell laid atop a flat body of water may develop wrinkles as means to overcome the geometric incompatibility. We denote by *D* the typical size of the shell and by *R* its radius of curvature. (**b**) We choose coordinates {*u*, *v*}, which at large scales follow the Cartesian coordinates {*x*, *y*} of the projection, however, at sub-wrinkle scales are proportional to arclength coordinates on the sheet. The amplitude *a*(*u*, *v*) and wave vector 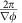 (written as in terms of a phase field *ϕ*(*u*, *v*)) may significantly vary across the sheet, however, the wavelength 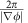 has a universal preferred value *λ*_0_. (**c**) Experiment: a hexagonal *D*=6 cm patch was cut out of a 30 μm-thick PDMS spherical shell, *R*=6 cm, before laid atop water. Shown is a top view schlieren image of the equilibrium configuration. (**d**) Simulation: a finite-element code with the physical parameters of the experiment (**c**). Shown is the vertical displacement from the plane. A single wrinkle (solid line) may bend, that is, change its in-plane direction, with wrinkle curvature encoded in the second derivatives of the phase field *ϕ*. In the shown example, the obtained pattern breaks into six domains, such as the lightened region on the right. Wrinkle bending focuses into domain walls (thicker solid segment of the wrinkle line). We denote by *L* the typical domain size. (**e**) The height squared profile of (**d**), at two different slices (horizontal and vertical), normalized by 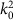. The envelope of these curves is by definition Δ(*u*, *v*). The black curve, 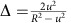, plotted for *R*=6 cm (no fitted parameters), is anticipated for an axisymmetric pattern. (**f**) The full Δ(*u*, *v*) measured from (**d**). (**g**) Sketch of the phase field *ϕ*(*u*, *v*) for the pattern in (**c**,**d**). Level sets of *ϕ* indicate wrinkles.

**Figure 3:**
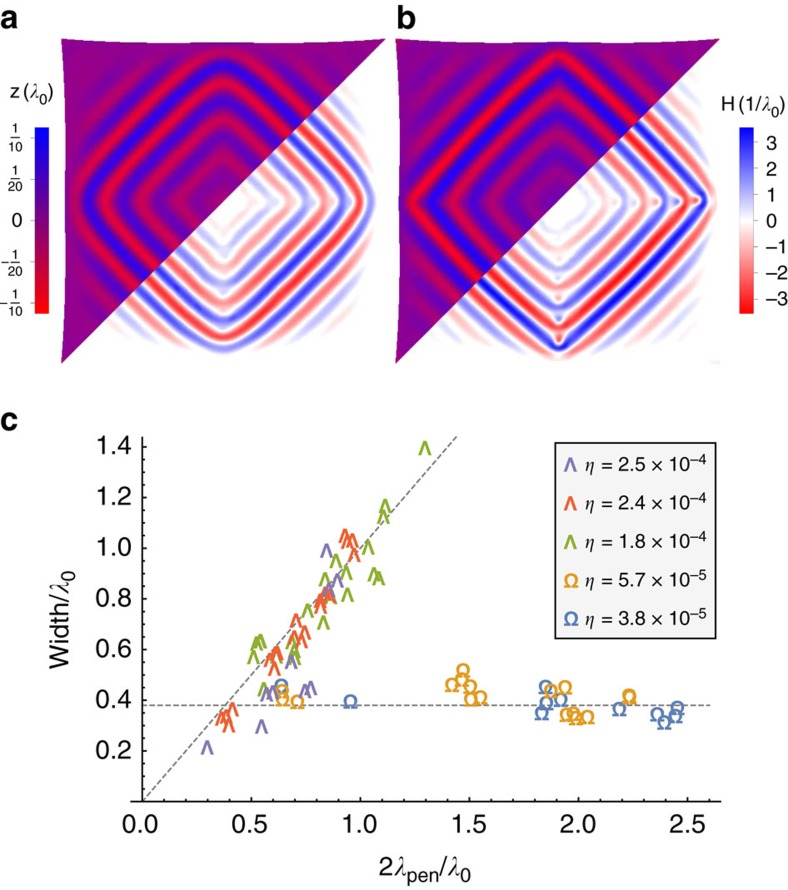
The width of domain walls. (**a**,**b**) Equilibrium wrinkle patterns in a square section of a spherical shell on liquid substrate (simulation). Vertical displacement *z* (top-left) and mean curvature *H* (bottom-right) clearly indicate splitting of the patterns into four domains divided by domain walls of varying widths. (**a**) *η*≈2.4 × 10^−4^, all domain walls are of the ‘Chevron’ type, that is, wrinkle angle changes monotonically when moving along the wrinkle across a domain wall. (**b**) *η*≈5.7 × 10^−5^, domain walls in the high-amplitude wrinkles become Ω-shaped through the formation of localized d-cones (cusp is well seen in the mean curvature map). (**c**) The width of Chevron-shaped domain walls is proportional to the penetration length (equation (18)), hence to the amplitude. Ω-shaped walls have widths proportional to the wrinkle wavelength, regardless of the amplitude. Widths are extracted from simulations by fitting wrinkle angle to an arctangent, in compliance with^19^. Penetration lengths are calculated from system parameters and the measured amplitude of each wrinkle.

**Figure 4:**
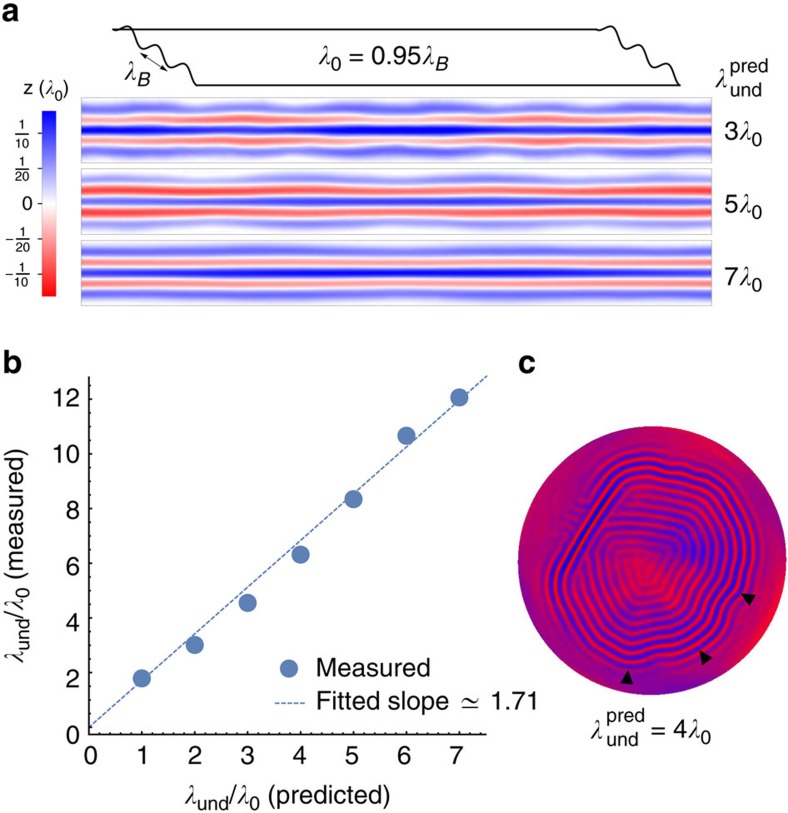
Undulation instability. (**a**) Equilibrium wrinkle patterns in a flat thin ribbon residing on a flat body of water (simulation), held at its far ends with a fixed wavy boundary. The preferred wrinkle wavelength *λ*_0_ is 5% shorter than the boundary wavelength *λ*_*B*_. Shown are the results for various values of the predicted undulation wavelength 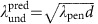 (equation (19)), where the ribbon’s width is used as a surrogate for the system scale *d*. (**b**) The measured undulation wavelength is plotted against the predicted one. While corroborating the predicted scaling, the measured wavelength is consistently larger than 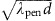 by a factor of ≈1.7, suggesting that the relevant system scale *d* may be different than the ribbon width by a constant factor. (**c**) Simulation results for a spherical cap with free boundary conditions. An undulation appears spontaneously, and its observed wavelength (black triangles) agrees with the predicted value, where the shell’s diameter appears to be a good surrogate for the system scale *d*.

We further observe that wrinkle undulations spontaneously appear in certain regions of larger systems with free boundary conditions as a result of the global geometric incompatibility. We show examples for this observation in Fig. 4c and in Supplementary Fig. 1. We conjecture that this instability sets the scale for the herringbone patterns seen in biaxially compressed sheets on elastic substrates^25^,^29^,^30^, as shown in Fig. 1d. The scaling implied by this conjecture agrees with the stability analysis scaling presented in ref. 31.

### Domain size scaling

Last, our smectic analogy approach can further give a coarse prediction for the typical size and number of domains in systems that wrinkle as a result of geometric incompatibility. The coarse-grained metric **g**^(cg)^ of a domain-type patterns is approximately that of a piecewise-linear configuration that approximates the curved target metric 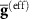. The strain that results from the difference between the two induces the elastic energy (16), therefore preferring smaller domains (that is, a better piecewise-linear approximation). However, the smectic energy term (17) is concentrated into domain walls, thus penalizing many-domain solutions. The interplay between these two contributions sets the typical domain size.

**Figure 5:**
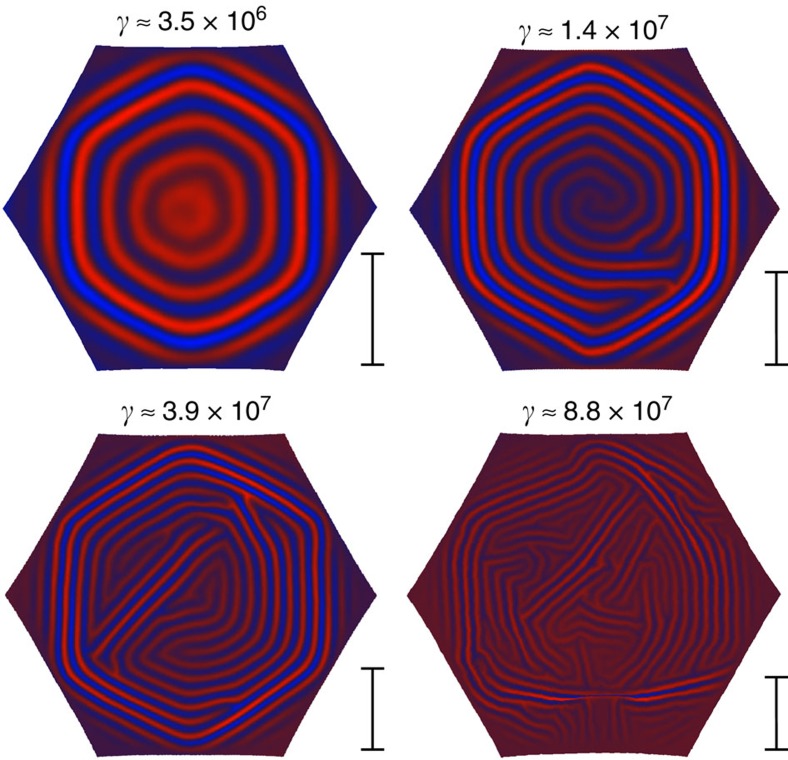
Domain size scaling. Typical equilibrium wrinkle patterns in a hexagonal section of a thin spherical shell on a liquid substrate. All simulation parameters are kept fixed, except for the sheet thickness. The Föppl–von Kármán number *γ* of the system is indicated above each pattern. While the top-left pattern is clearly divided into six domains, increasing *γ* seems to decrease the typical domain size. The scale bar to the right of each pattern shows the scaling of typical domain sizes predicted by equation (20), up to a constant factor (only the ratios between bars are meaningful since equation (20) suggests only the scaling of the typical size and leaves the prefactor unknown).

We consider a spherical shell with radius of curvature *R*. For domains of typical size *L*, the maximal (average) elastic strain scales as ∝ *L*^2^*R*^−2^ (*R*^−2^ is the Gaussian curvature mismatch). Integrated over the entire sheet of typical size *D*, this will result in total elastic energy 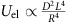. The energy per-unit-length of a domain wall is proportional to the smectic curvature modulus divided by the smectic penetration length^19^, that is, scales as ∝Δ^3/2^*η*^1/2^*λ*_0_∝Δ^3/2^*t*. Upon introducing the typical excess length implied by the global geometric incompatibility, 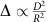, and the total length of the walls ∝*D*^2^/*L*, we get the total smectic contribution 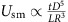. Minimization of the total energy (15) suggests the scaling


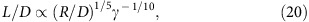


where *γ* is the Föppl–von Kármán number. This general scaling relation should be taken with caution, since the number of domains is discrete and is very much affected by boundary conditions. A quantitative experimental or numerical verification of equation (20) must therefore involve very large systems (so that boundary effects are irrelevant), with typical domain size extracted via statistical analyses of the pattern. Another experimental complication involves the very small exponent by which the domain size in equation (20) depends on physical parameters. Preliminary numerical observations may support relation (20) (Fig. 5), however, a robust numerical verification using a finite-element thin-sheet simulation is computationally inconceivable as a result of the extreme multi-scale nature of the problem. Nevertheless, we hope that a more efficient simulation based on our phase field *ϕ* approach may in the future allow verification of this scaling.

## Discussion

We have shown that thin elastic systems that develop wrinkled patterns share similar physics to that of smectic liquid crystals and other stripe-patterned systems over a wide parameter regime. The analogy allows us to make quantitative predictions regarding several aspects of wrinkled patterns. Not less importantly, it sheds new light on the behaviour of such systems, and extends our intuition about the emerging patterns observed in them. We show numerically that behaviour typical to smectic systems is indeed evident in wrinkled patterns.

Our theory relies on several key assumptions, primarily the scale separation between the wrinkle wavelength and other scales. These include not only controlled system parameters such as the sheet dimensions or intrinsic geometry radii of curvature, but also emergent length scales such as the amplitude or the variation in the pattern’s envelope. These assumptions hold globally in many systems, as we showed in this paper. In other parameter regimes these assumptions break, with an apparent tendency to localize into small regions in the form of deep folds or creases. These localized structures cannot be accounted for by including higher orders in the small parameters we introduced. Their geometrical features as well as their energetic contribution must be estimated non-perturbatively to understand when the formation of such structures is favourable—fodder for future work.

In addition, if one wishes to predict more precisely the emergent patterns, and not just their general features and bulk statistics, it is necessary to formulate the effective boundary conditions for the smectic model. These rules may be complicated as they involve both the smectic phase field and the residual elastic strain field coupled together and will, in many cases, involve the appearance of a non-wrinkled flattened boundary layer^32^. They are therefore in need of further investigation. Formulating these boundary conditions and combining them with the bulk equations presented in this paper will complete the description of wrinkled systems on soft substrates in terms of only smooth fields in the 2D Euclidean plane. It may therefore be a basis for simple and efficient numerical simulations of these systems that will allow further investigation of their rich pattern phenomenology.

### Data availability

The data that support the findings of this study are available from the corresponding authors upon reasonable request.

## Additional information

**How to cite this article:** Aharoni, H. *et al*. The smectic order of wrinkles. *Nat. Commun.* **8,** 15809 doi: 10.1038/ncomms15809 (2017).

**Publisher’s note**: Springer Nature remains neutral with regard to jurisdictional claims in published maps and institutional affiliations.

## Supplementary Material

Supplementary Information
