# Supplementary material for: The smectic order of wrinkles
Source: Nat Commun. 2017 Jul 18;8:15809. doi: 10.1038/ncomms15809 (PMC5520158; doi:10.1038/ncomms15809)
Supplement: Supplementary Information [file ncomms15809-s1.pdf]

Type of file: PDF
Size of file: 0 KB
Title of file for HTML: Supplementary Information
Description: Supplementary Note and Supplementary Figures

Supplementary Information

Supplementary Note 1. Derivation of the Elastic/Smectic Energy Density

In the main text we describe the coarse-grained energy density in the (u, v) coordinates. It reads

$$U^{(\mathrm{cg})} \sim \frac{Y}{8}\left\|\mathbf{g}^{(\mathrm{cg})}-\bar{\mathbf{g}}\right\|^2+\frac{B}{2}\left\|\bar{\mathbf{b}}\right\|^2+\frac{Y\Delta^2}{256}\left(\frac{\|\nabla\nabla\phi\|}{|\nabla\phi|^2}\right)^2+\frac{B\Delta}{4}|\nabla\phi|^2+\frac{K\Delta}{4|\nabla\phi|^2}, \tag{1}$$

where $\sim$ indicates that we have omitted higher-order terms in the small parameters $\frac{\|\nabla\nabla\phi\|}{|\nabla\phi|^2}$, $\frac{|\nabla a|}{a|\nabla\phi|}$ and Δ. The only difference between supplementary equation (1) and main text equation (14) is that the amplitude a was everywhere replaced with Δ according to the latter's definition, $\Delta \equiv a^2|\nabla\phi|^2$. Our motivation for making this substitution is that while a and $|\nabla\phi|$ may be changed locally without changing the sheet's $2D$ geometry, their product $\sqrt{\Delta}$ may not as it directly reflects lengths on the sheet (see main text equation (5)). Any change from the sheet's intrinsic $2D$ geometry causes elastic stretching, which is energetically unfavorable for a thin sheet. Formally, we notice that by the triangle inequality

$$\left|\frac{\Delta}{2}-\|\bar{\mathbf{g}}-\mathbf{I}\|\right| = \left|\|\mathbf{g}^{(\mathrm{cg})}-\mathbf{I}\|-\|\bar{\mathbf{g}}-\mathbf{I}\|\right| \leq \left\|\mathbf{g}^{(\mathrm{cg})}-\bar{\mathbf{g}}\right\|. \tag{2}$$

We expect the right-hand side of supplementary equation (2) to be small since it is coupled to the stretching modulus Y, which is, under our assumptions, much larger than any other modulus in supplementary equation (1). The left-hand side of supplementary equation (2) must therefore also be small, hence Δ is set by the difference between the reference metric and the flat Euclidean metric of the projection.

We now turn to rewrite the last two terms of supplementary equation (1) in the form of an expansion in $|\nabla\phi|$ around the balance between these two terms. We again use the fact that Δ is set independently of $|\nabla\phi|$ at every point to write

$$\begin{aligned}\frac{B\Delta}{4}|\nabla\phi|^2+\frac{K\Delta}{4|\nabla\phi|^2} &= \frac{\sqrt{BK}\Delta}{4}\left(\frac{|\nabla\phi|^2}{\sqrt{K/B}}+\frac{\sqrt{K/B}}{|\nabla\phi|^2}\right) = \\ &= \frac{Y\eta\Delta}{4}\left(\frac{|\nabla\phi|^2}{{k_0}^2}+\frac{{k_0}^2}{|\nabla\phi|^2}\right) = \\ &= \frac{Y\eta\Delta}{4}\left[2+\left(\frac{|\nabla\phi|^2}{{k_0}^2}-1\right)^2+O\left(\left|\frac{|\nabla\phi|^2}{{k_0}^2}-1\right|^3\right)\right] = \\ &= \frac{Y\eta\Delta}{2}+\frac{Y\eta\Delta}{4}\left(\frac{|\nabla\phi|^2}{{k_0}^2}-1\right)^2+O\left(\left|\frac{|\nabla\phi|^2}{{k_0}^2}-1\right|^3\right).\end{aligned} \tag{3}$$

Plugging supplementary equation (3) back into supplementary equation (1) we get

$$U^{(\mathrm{cg})} = \underbrace{\frac{Y}{8}\left\|\mathbf{g}^{(\mathrm{cg})}-\bar{\mathbf{g}}\right\|^2+\frac{Y\eta\Delta}{2}+\frac{B}{2}\left\|\bar{\mathbf{b}}\right\|^2}_{U_{\mathrm{elastic}}}+\underbrace{\frac{Y\eta\Delta}{4}\left(\frac{|\nabla\phi|^2}{{k_0}^2}-1\right)^2+\frac{Y\Delta^2}{256}\left(\frac{\|\nabla\nabla\phi\|}{|\nabla\phi|^2}\right)^2}_{U_{\mathrm{smectic}}}+\text{h.o.t.}, \tag{4}$$

where "h.o.t" now include also higher order in the smectic compression $\left||\nabla\phi|^2/{k_0}^2-1\right|$, as those terms are higher order in the small parameters via our above arguments.

In addition, one gets a self-consistent correction to the reference metric by substituting $\Delta = 2\|\mathbf{g}^{(\mathrm{cg})}-I\|$ back into the elastic term in supplementary equation (4). Interpreted in this manner, one sees that not only deviations of $\mathbf{g}^{(\mathrm{cg})}$ from the reference metric $\bar{\mathbf{g}}$ are penalized, but also, to a much lesser extent set by the small parameter $\eta = \sqrt{BK}/Y$, deviations of $\mathbf{g}^{(\mathrm{cg})}$ from the flat metric $\mathbf{I}$. These two terms can then be rewritten, expanded around their combined minimizer $\bar{\mathbf{g}}^{(\mathrm{eff})} = \bar{\mathbf{g}} - 4\eta\frac{\bar{\mathbf{g}}-\mathbf{I}}{\|\bar{\mathbf{g}}-\mathbf{I}\|}$, to get the form that appears in main text equation (16).

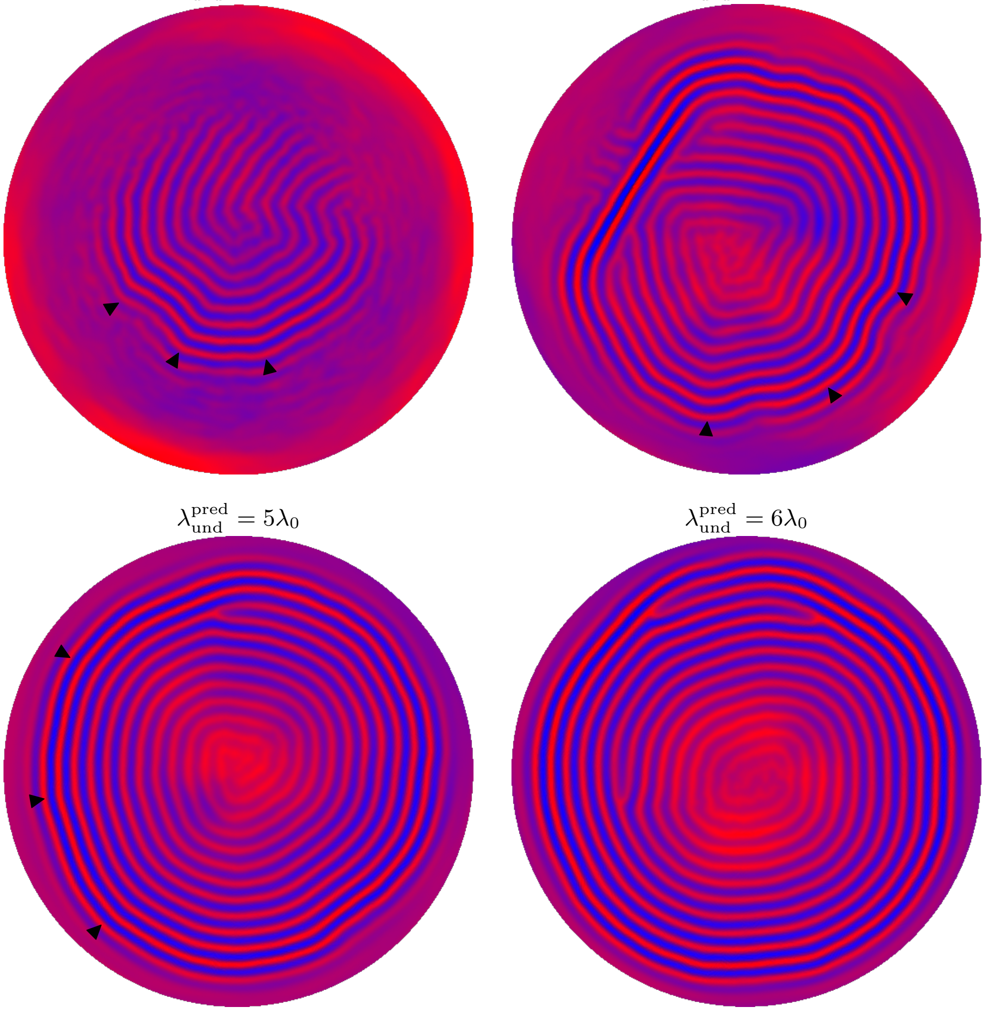

Supplementary Figure 1: Undulation instability in free boundary patterns. Typical equilibrium wrinkle patterns in a simulated circular section of a spherical shell residing on a flat body of water. The sheet thickness and water density are tweaked so that the typical wrinkle wavelength λ_0 remains constant at $D/\lambda_0 = 30$, where D is the shell diameter, however the predicted undulation instability wavelength $\lambda_{\rm und}^{\rm pred} = \sqrt{\lambda_{\rm pen} D}$ is varied. Shown equilibria are not necessarily the global ground states, nonetheless the typical visible undulation wavelength appears independent of initial conditions. Black triangles are guide to the eye, to highlight this typical undulation. The shell diameter D appears to be a good surrogate for the "system scale" d in main text eq. (19).

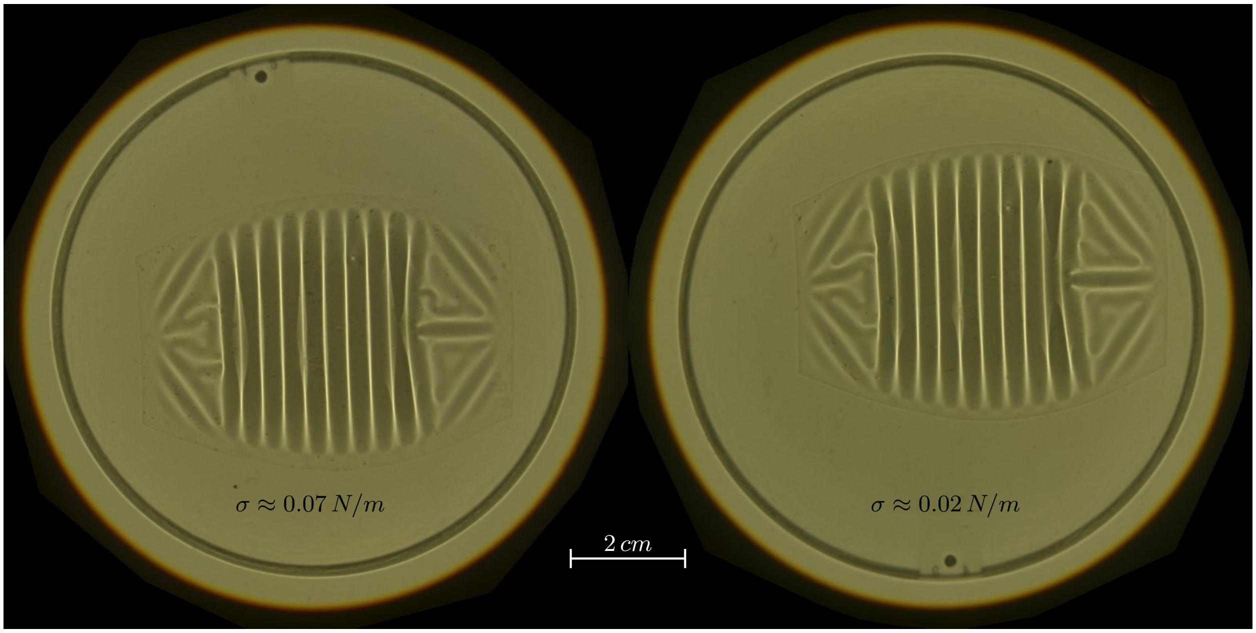

Supplementary Figure 2: Insignificant effect of surface tension. Equilibrium wrinkle patterns obtained in experiment for a rectangular section of a spherical cap residing on a flat fluid substrate. The stretching modulus of the $30\mu m$-thick sheet is $Y \approx 35\,N/m$. Initially, pure water with surface tension $\sigma \approx 0.07\,N/m$ was used as a substrate (left). Soap was then gradually introduced to the water, until reaching a solution with surface tension $\sigma \approx 0.02\,N/m$ (right). As can be seen, no significant change in the equilibrium patterns is observed. This follows because the geometric compatibility already induces tension on the boundary of the shell, as can be seen by the vanishing amplitude. A very careful examination shows that this flattened region does slightly narrow upon lowering the surface tension. However, since surface tension is orders of magnitude lower then the stretching modulus, this is a very small effect and is not significant enough to change patterns in the bulk.
